# Clinical factors associated with acute abdominal symptoms induced by gastric anisakiasis: a multicenter retrospective cohort study

**DOI:** 10.1186/s12876-023-02880-7

**Published:** 2023-07-18

**Authors:** Yutaka Okagawa, Tetsuya Sumiyoshi, Takayuki Imagawa, Hiroya Sakano, Fumito Tamura, Yohei Arihara, Yusuke Kanari, Akira Sakurada, Shutaro Oiwa, Takashi Jin, Yusuke Tomita, Shinya Minami, Hiroyuki Hisai, Hirohito Muramatsu, Shinichi Katsuki, Masahiro Maeda, Hitoshi Kondo

**Affiliations:** 1 Department of Gastroenterology, Tonan Hospital, North 4, West 7, Chuo-ku, 060-0004 Sapporo, Hokkaido Japan; 2 Department of Gastroenterology, Rumoi Municipal Hospital, Rumoi, Hokkaido Japan; 3 Department of Gastroenterology, Japanese Red Cross Date Hospital, Date, Hokkaido Japan; 4 Department of Gastroenterology, Sapporo Kiyota Hospital, Sapporo, Hokkaido Japan; 5 Department of Gastroenterology, Steel Memorial Muroran Hospital, Muroran, Hokkaido Japan; 6 Department of Gastroenterology, Chitose City Hospital, Chitose, Hokkaido Japan; 7 Department of Gastroenterology, Otaru Ekisaikai Hospital, Otaru, Hokkaido Japan; 8 Department of Gastroenterology, Oji General Hospital, Tomakomai, Hokkaido Japan; 9 Department of Internal Medicine, Rishiri Island National Health Insurance Center Hospital, Rishiri, Hokkaido Japan

**Keywords:** Gastric anisakiasis, Abdominal pain, Asymptomatic

## Abstract

**Background:**

Gastric anisakiasis typically causes severe abdominal symptoms; however, we incidentally detected asymptomatic gastric anisakiasis cases during esophagogastroduodenoscopy. The factors associated with developing acute abdominal symptoms induced by gastric anisakiasis remain unclear. Therefore, this study aimed to investigate the clinical factors associated with abdominal symptoms of gastric anisakiasis by comparing symptomatic and asymptomatic cases.

**Methods:**

This was a retrospective cohort study involving 264 patients diagnosed with gastric anisakiasis at nine hospitals in Japan between October 2015 and October 2021. We analyzed patients’ medical records and endoscopic images and compared the clinical factors between the symptomatic and asymptomatic groups.

**Results:**

One hundred sixty-five patients (77.8%) were diagnosed with abdominal symptoms, whereas 47 (22.2%) were asymptomatic. Older age, male sex, diabetes mellitus, gastric mucosal atrophy, and gastric mucosal atrophy of the *Anisakis* penetrating area were significantly more common in the asymptomatic group than in the symptomatic group. Multivariate analysis revealed that age (*p* = 0.007), sex (*p* = 0.017), and presence or absence of mucosal atrophy (*p* = 0.033) were independent factors for the occurrence of acute abdominal symptoms. In addition, cases that were *Helicobacter pylori* naïve, with an elevation of white blood cells, or without an elevation of eosinophils were more common in the symptomatic group than in the asymptomatic group.

**Conclusions:**

Age, sex, and presence or absence of gastric mucosal atrophy were the clinical factors associated with the occurrence of acute abdominal symptoms. Older and male patients and those with gastric mucosal atrophy were less likely to show abdominal symptoms. The mechanisms of the occurrence of symptoms induced by gastric anisakiasis remain unclear; however, our results will help clarify this issue in the future.

## Introduction

Gastrointestinal anisakiasis is a zoonotic parasitic infection caused by ingesting raw or uncooked seafood infected with nematodes of the genus *Anisakis*. *Anisakis simplex* is the most common cause of the infection [1, 2]. Most cases of anisakiasis are reported from Japan, with approximately 20,000 cases occurring yearly [3], possibly due to the Japanese culture of ingesting raw fish. In recent years, Japanese foods (sushi and sashimi) have become popular worldwide and are expected to cause an increased incidence of gastrointestinal anisakiasis [4]. There have been reports of gastrointestinal anisakiasis in various countries and regions [5, 6], and this disease has been recognized as a public health concern. *Anisakis* larvae may parasitize the esophagus, stomach, small bowel, and colon; however, most gastrointestinal anisakiasis cases are gastric anisakiasis, representing approximately 95% of cases [7]. Gastrointestinal anisakiasis is characterized by an acute abdomen, and the typical symptom of gastric anisakiasis is acute severe epigastric pain with a few hours after ingesting infected seafood. Other symptoms may include nausea and vomiting. Symptoms usually develop within 48 h (peaking within 6 h) [8].

Gastric anisakiasis is generally thought to cause severe abdominal symptoms; however, we incidentally detected asymptomatic gastric anisakiasis cases during esophagogastroduodenoscopy (EGD) for screening or medical checkups. To date, only a few case reports of asymptomatic gastric anisakiasis exist [9, 10]; however, such cases are sometimes encountered in actual clinical practice. Factors associated with developing abdominal symptoms induced by gastric anisakiasis remain unclear. A previous study showed that *Anisakis simplex* tended to penetrate non-atrophic mucosa more than atrophic mucosa, and patients with normal mucosal infections were more likely to exhibit clinical symptoms [11]. Gastric mucosal atrophy is yellowish-pale and unsmooth mucosa mainly associated with *Helicobacter pylori (H. pylori)* infection. Another group reported an association between clinical manifestations and *H. pylori* infection [12]. However, these studies were conducted at a single center with a small sample size and predominantly symptomatic cases; no study analyzing the clinical factors between symptomatic and asymptomatic cases in a multicenter setting with a large sample size exists. Therefore, we conducted a multicenter retrospective cohort study to investigate factors associated with acute abdominal symptoms induced by gastric anisakiasis.

## Materials and methods

### Study design

This was a multicenter, retrospective cohort study conducted at nine hospitals in Japan, in compliance with the principles of the Declaration of Helsinki of 1964 and revised versions. The study protocol was approved by the institutional review board of Tonan Hospital (approval number 547) and all participating institutions. Written informed consent for EGD was obtained from all the patients. All participants were given opportunities to decline participation in this study using the opt-out method on each participating hospital’s website. This study was registered on the University Hospital Medical Information Network (Registration number UMIN 000046411).

### Patients

This study included consecutive patients diagnosed with gastric anisakiasis at nine hospitals in Japan between October 2015 and October 2021. Patients who were reinfected with *Anisakis* within the period were also included. The inclusion criteria were cases aged ≥ 20 years, diagnosed with gastric anisakiasis using EGD, and wherein *Anisakis* larvae were removed with forceps. Gastric anisakiasis was defined as *Anisakis* larvae penetrating into the gastric wall. The exclusion criteria were clinical symptoms or endoscopic findings that could not be evaluated, and non-provision of clinical information. Patients of multiple *Anisakis* larvae infection (two or more *Anisakis* larvae found concurrently) were also excluded because it was impossible to determine which larvae were causing symptoms.

### Outcome measures

We analyzed patients’ medical records and endoscopic images, including background, comorbidities, regular use of non-steroidal anti-inflammatory drugs (NSAIDs) or acid secretion inhibitors, degree of gastric mucosal atrophy, location of *Anisakis* larvae, gastric mucosal atrophy of *Anisakis* larvae’s penetrating area, surrounding edema, erythema or erosion, and complications associated with the endoscopic procedure. If available, we also analyzed the *H. pylori* infection status and laboratory data, including white blood cell (WBC) counts (cut off < 8600/µL), the percentage of eosinophils (cut off < 6%), and C-reactive protein (CRP) level (cut off < 0.14 mg/dL). The degree of gastric mucosal atrophy was assessed using the Kimura-Takemoto classification [13]. This classification categorized the extent of atrophy into closed-type (C1, C2, and C3) and open-type (O1, O2, and O3). C0 indicated no atrophic mucosa, whereas C1 to O3 indicated atrophic mucosa. In this study, we analyzed whether there was a difference in the occurrence of symptoms depending on the degree of mucosal atrophy. The presence or absence of mucosal atrophy of *Anisakis* larvae penetrating area was defined as follows: an endoscope that showed homogeneously reddish and smooth mucosa with regular arrangement of collecting venules (RAC) indicated no atrophy, whereas yellowish-pale mucosa and unsmooth mucosa without RAC indicated the presence of atrophy [14]. Gastric mucosal atrophy, mucosal edema, erythema, or erosion were evaluated by endoscopic images. *H. pylori* infection status was divided into three groups. If any of the tests (urea breath, rapid urease, stool antigen, or serum *H. pylori* immunoglobulin G antibody test) were positive, the patient was considered to have *H. pylori* infection. If the tests were negative and the patient had a positive history of eradication, or if any of the tests were negative and endoscopic findings showed mucosal atrophy, the patient was considered to have *H. pylori* eradication. If one or more of these tests were negative and endoscopic findings showed C0, the patient was considered *H. pylori* naïve. We categorized patients into two groups according to their clinical symptoms: (a) symptomatic group: patients with acute abdominal pain with or without nausea, or vomiting; and (b) asymptomatic group: patients without abdominal symptoms, such as those incidentally detected during EGD for screening or medical checkups. We compared the clinical findings between the two groups to investigate the clinical factors associated with the occurrence of acute abdominal symptoms.

### Statistical analysis

All statistical analyses were performed using EZR (Saitama Medical Center, Jichi Medical University, Saitama, Japan), which is a graphical user interface for R 2.13.0 (R Foundation for Statistical Computing, Vienna, Austria) [15]. Quantitative variables were expressed as medians, whereas categorical variables were presented as total numbers and percentages. Pearson’s chi-squared and the Mann-Whitney U tests were used as appropriate. Multivariate analysis was performed using a logistic regression analysis. Statistical significance was set at *p* < 0.05.

## Results

### Patients’ characteristics

**Fig. 1:**
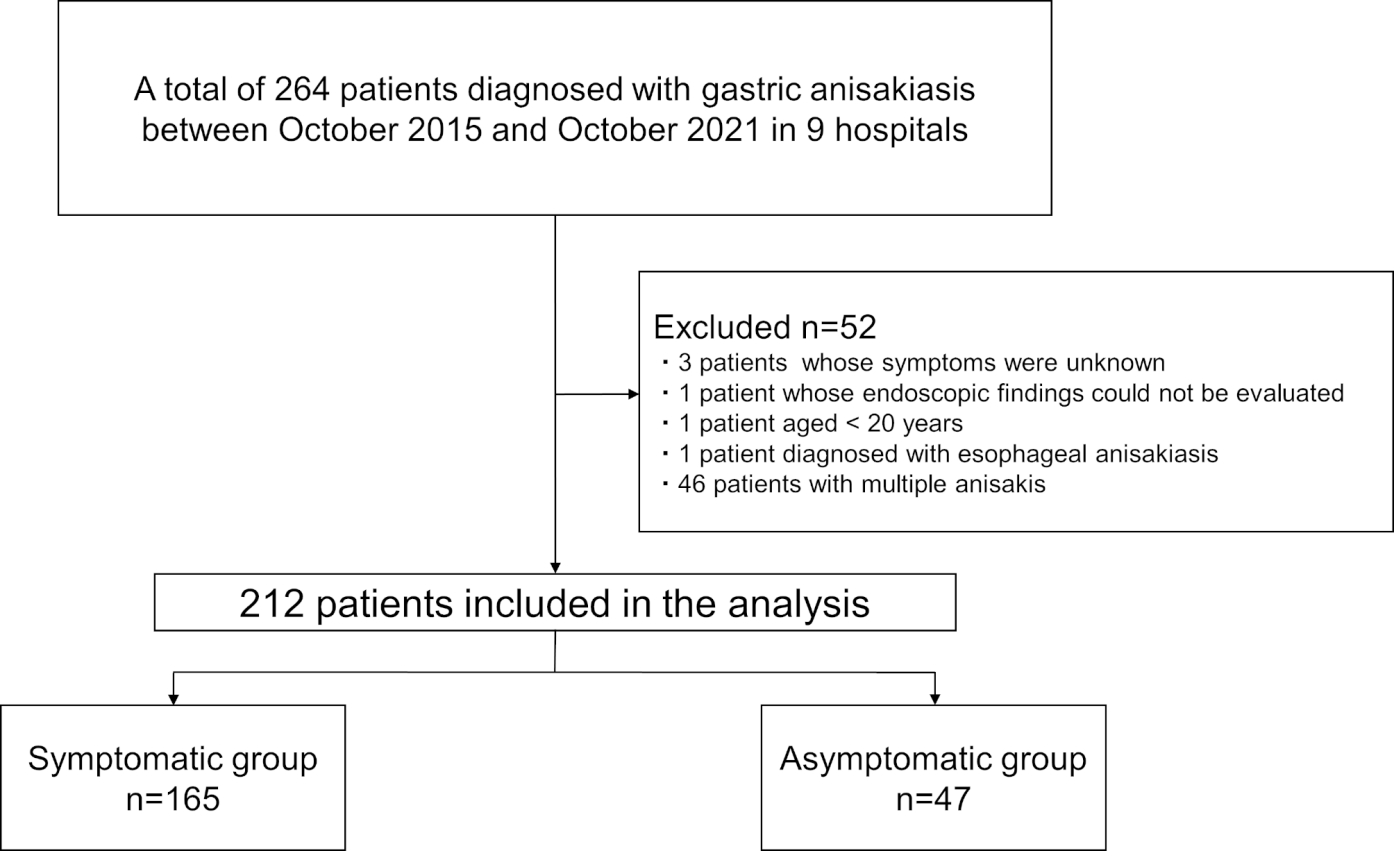
Flow chart outlining the selection of patients

**Fig. 2:**
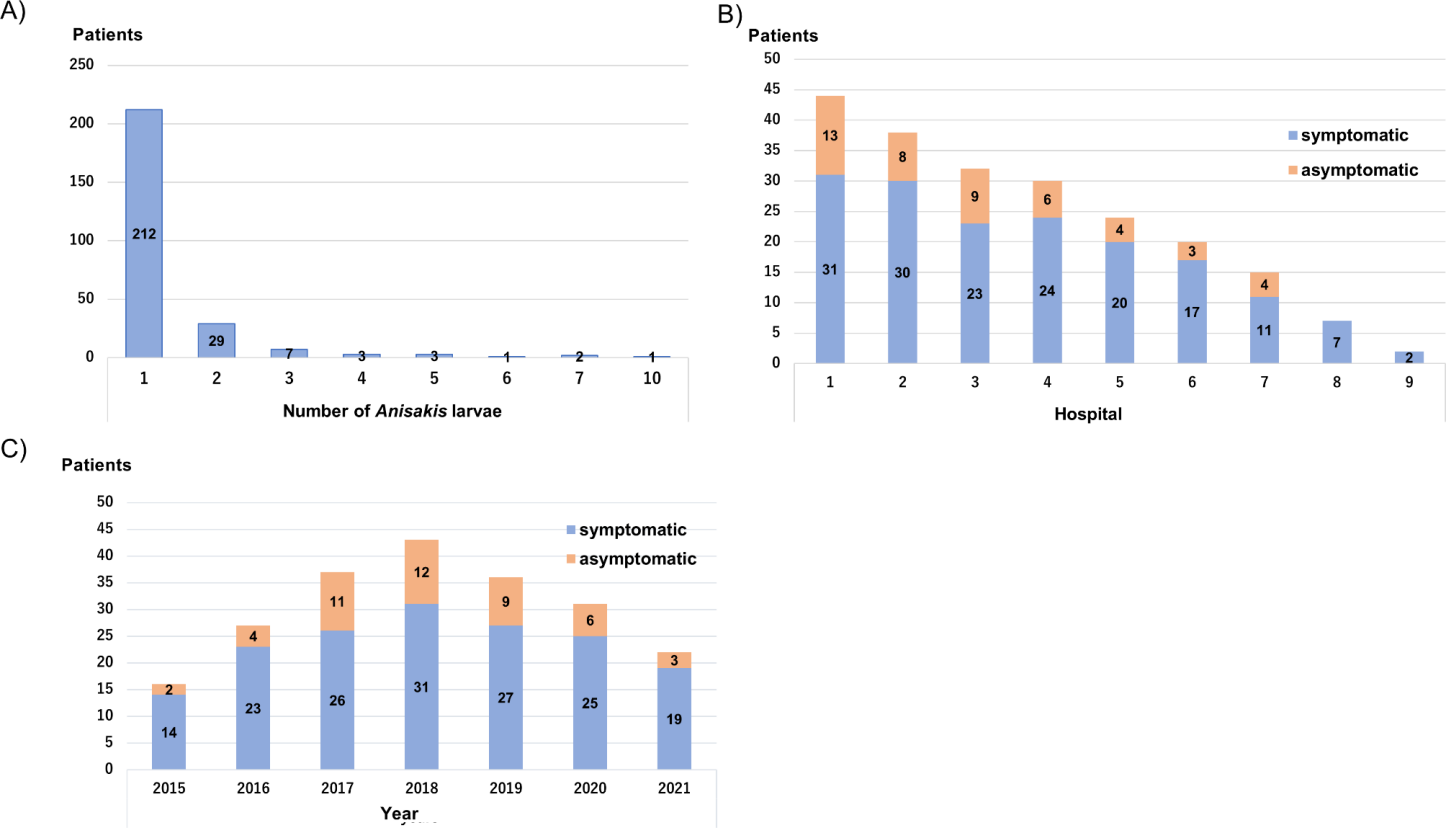
**A**) Number of patients and number of *Anisakis* larvae. **B**) Number of patients per hospital. **C**) Number of patients per year

A total of 264 patients diagnosed with gastric anisakiasis at nine hospitals between October 2015 and October 2021 were enrolled in this study. Of the 264 patients, 52 were excluded for the following reasons: unknown clinical symptoms (n = 3), inability to evaluate endoscopic findings (n = 1), age < 20 years (n = 1), esophageal anisakiasis (n = 1), and multiple *Anisakis* larvae infections (n = 46). Therefore, 212 patients were finally analyzed (Fig. 1). Patients’ baseline characteristics are presented in Table 1. There were 116 male and 96 female patients, with a median patient age of 53 years. Of all the patients, 7.1% had diabetes mellitus. Furthermore, 3.3% and 17.5% of patients took NSAIDs and acid secretion inhibitors, respectively. One hundred sixty-five patients (77.8%) were diagnosed with abdominal symptoms, whereas 47 (22.2%) were asymptomatic. According to the classification of background gastric mucosal atrophy, 145 patients (68.4%) were classified as C0, 46 (21.7%) as C1–C3, and 21 (9.9%) as O1–O3. The locations of *Anisakis* larvae were as follows: the upper third (n = 74, 34.9%), middle third (n = 96, 45.3%), and lower third (n = 42, 19.8%) of the stomach. According to the gastric circumference, 30 larvae were detected in the lesser curvature (14.2%), 123 in the greater curvature (58.0%), 25 in the anterior wall (11.8%), and 34 in the posterior wall (16.0%). The presence of atrophy in the *Anisakis* larvae’s penetrating area was positive in 43 (20.3%) patients. Surrounding edema, erythema, or erosion was positive in 164 (77.4%) lesions. No complications were associated with the endoscopic procedures. In this study, no cases reinfected with *Anisakis* were observed within the period. Detailed data about the number of *Anisakis* larvae, the number of patients per hospital, and the number of patients per year are shown in Fig. 2.

**Table 1:** Patient characteristics

	n = 212 (%)
Age (years)	
Median (range)	53 (21–89)
Sex	
Male	116 (54.7)
Female	96 (45.3)
Abdominal pain	
Present	165 (77.8)
Absent	47 (22.2)
Comorbidities	
Ischemic heart disease	10 (4.7)
Liver cirrhosis	2 (0.9)
Diabetes mellitus	15 (7.1)
CKD with dialysis	1 (0.5)
NSAIDs	7 (3.3)
Acid secretion inhibitor	37 (17.5)
Degree of gastric mucosal atrophy	
No atrophy	145 (68.4)
Closed type	46 (21.7)
Open type	21 (9.9)
Location	
Upper third	74 (34.9)
Middle third	96 (45.3)
Lower third	42 (19.8)
Circumference	
Anterior wall	25 (11.8)
Posterior wall	34 (16.0)
Greater curvature	123 (58.0)
Lesser curvature	30 (14.2)
Mucosa of penetrating area	
Atrophy	43 (20.3)
No atrophy	169 (79.7)
Edema, erythema, erosion	
Positive	164 (77.4)
Negative	48 (22.6)

CKD; chronic kidney disease, NSAIDs; non-steroidal anti-inflammatory drugs

### Comparison of clinical findings between symptomatic and asymptomatic groups

A comparison of the clinical findings between the symptomatic and asymptomatic groups is shown in Table 2. The median age was 49 years in the symptomatic group and 64 years in the asymptomatic group, indicating that the patients in the asymptomatic group were significantly older (*p* < 0.001). The number of female patients in the symptomatic group was significantly higher than that in the asymptomatic group (*p* = 0.046). Furthermore, there were significant differences in the presence of liver cirrhosis (*p* = 0.048) and diabetes mellitus (*p* = 0.026) between the two groups. In contrast, the administration of NSAIDs or acid secretion inhibitors was not significantly different between the two groups. Regarding the degree of mucosal atrophy, 75.2% and 44.7% of patients were classified as having no atrophic mucosa in the symptomatic and asymptomatic groups, respectively, with a significant difference (*p* = 0.001). However, there was no significant difference in the penetrating location or circumference of *Anisakis*, and the larvae were more common in the greater curvature of the upper or middle stomach. The presence of atrophy in the *Anisakis* larvae penetrating area was positive in 17.0% of patients in the symptomatic group and 31.9% of those in the asymptomatic group, with a significant difference (*p* = 0.038). There was no significant difference in the presence of surrounding erosion, edema, or erythema between the two groups.

**Table 2:** Comparison of clinical findings between symptomatic and asymptomatic groups

	Symptomatic group	Asymptomatic group	
	n = 165 (%)	n = 47 (%)	p value
Age (years)			
Median (range)	49 (21–82)	64 (38–89)	< 0.001
Sex			0.046
Male	84 (50.9)	32 (68.1)	
Female	81 (49.1)	15 (31.9)	
Comorbidities			
Ischemic heart disease	7 (4.2)	3 (6.4)	0.700
Liver cirrhosis	0 (0.0)	2 (4.3)	0.048
Diabetes mellitus	8 (3.8)	7 (14.9)	0.026
CKD with dialysis	0 (0.0)	1 (2.1)	0.220
NSAIDs	6 (3.6)	1 (2.1)	0.874
Acid secretion inhibitor	25 (15.2)	12 (25.5)	0.126
Gastric mucosa			0.001
No atrophy	124 (75.2)	21 (44.7)	
Atrophy	41 (24.8)	26 (55.3)	
Location			0.386
Upper third	57 (34.5)	17 (36.2)	
Middle third	72 (43.6)	24 (51.1)	
Lower third	36 (21.8)	6 (12.8)	
Circumference			0.163
Anterior wall	15 (9.1)	10 (21.3)	
Posterior wall	28 (17.0)	6 (12.8)	
Greater curvature	97 (58.8)	26 (55.3)	
Lesser curvature	25 (15.2)	5 (10.6)	
Mucosa of penetrating area			0.038
Atrophy	28 (17.0)	15 (31.9)	
No atrophy	137 (83.0)	32 (68.1)	
Edema, erythema, erosion			0.429
Positive	130 (78.8)	34 (72.3)	
Negative	35 (21.2)	13 (27.7)	

CKD; chronic kidney disease, NSAIDs; non-steroidal anti-inflammatory drugs

Pearson’s chi-squared test, Mann-Whitney U test

We performed a multivariate analysis of factors associated with acute abdominal symptoms, including age, sex, diabetes mellitus, presence or absence of mucosal atrophy, and mucosal atrophy of the penetrating area (Table 3). Age (*p* = 0.007), sex (*p* = 0.017), and the presence or absence of mucosal atrophy (*p* = 0.033) were independent clinical factors for the occurrence of acute abdominal symptoms.

**Table 3:** Multivariate analysis of factors associated with acute abdominal symptoms

	odds ratio	95% CI	p value
**Age**	0.965	0.940–0.990	0.007
**Sex**	0.406	0.193–0.852	0.017
Diabetes mellitus	0.592	0.176–1.990	0.397
**Mucosal atrophy**	0.393	0.166–0.927	0.033
Mucosal atrophy of penetrating area	0.774	0.193–0.852	0.602

Logistic regression analysis

We analyzed atrophic mucosal cases to determine the relationship between the degree of mucosal atrophy and the occurrence of acute abdominal symptoms (Table 4). First, we divided atrophic mucosal cases into closed- and open- type and found that the degree of mucosal atrophy was significantly associated with the occurrence of symptoms (*p* = 0.037).

**Table 4:** Relationship between the degree of mucosal atrophy and the occurrence of abdominal symptoms

	Degree of mucosal atrophy		
	Closed type n = 46 (%)	Open type n = 21 (%)	p value
Symptomatic	32 (69.6)	9 (42.9)	0.037
Asymptomatic	14 (30.4)	12 (57.1)	

Pearson’s chi-squared test

- ***H. pylori*status and laboratory data**.

*H. pylori* infection status, WBC counts, the percentage of eosinophils, and CRP levels were examined in 98 (46.2%), 123 (58.0%), 92 (43.4%), and 106 (50.0%) patients, respectively (Table 5). Regarding the *H. pylori* status, 70 patients (42.4%) in the symptomatic group and 28 (59.6%) in the asymptomatic group were examined. There was a significantly higher proportion of *H. pylori*-naïve patients in the symptomatic group than in the asymptomatic group (*p* < 0.01). WBC counts were examined in 98 (59.4%) and 25 (53.2%) patients in the symptomatic and asymptomatic groups, respectively. In the symptomatic group, 50 patients (51.0%) had WBC counts above the cut-off level, whereas all patients in the asymptomatic group had WBC counts within the normal limit. There was a significant difference between the groups (*p* < 0.001). Regarding the percentage of eosinophils, 83 (50.3%) and 9 (19.1%) patients were examined in the symptomatic and asymptomatic groups, respectively. The results show that 95.2% and 66.6% of the patients in the symptomatic and asymptomatic groups, respectively, exhibited a normal percentage of eosinophils (*p* = 0.002). CRP levels were examined in 92 (55.8%) and 14 (29.8%) patients in the symptomatic and asymptomatic groups, respectively, and no significant difference was observed (*p* = 0.200).

**Table 5:** Comparison of *Helicobacter pylori* infection status and laboratory data between symptomatic and asymptomatic groups

	Symptomatic group	Asymptomatic group	p value
**Hp status** (n = 98)			0.007
Infected	7 (10.0%)	3 (10.7%)	
Eradicated	22 (31.4%)	18 (64.3%)	
Naïve	41 (58.6%)	7 (25.0%)	
**WBC** (n = 123)			
Median (range) (/µL)	8600 (1400–21,570)	5300 (2900–7800)	
W.N.L / over	48 / 50	25 / 0	< 0.001
**Eosinophils** (n = 92)			
Median (range) (%)	1.0 (0-16.5)	2.0 (0.4-8)	
W.N.L / over	79 / 4	6 / 3	0.002
**CRP** (n = 106)			
Median (range) (mg/dL)	0.2 (0-7.8)	0.0 (0-7.4)	
W.N.L / over	42 / 50	9 / 5	0.200

Hp; *Helicobacter pylori*, WBC; White blood cell, W.N.L; Within the normal limit, CRP; C-reactive protein

Pearson’s chi-squared test

## Discussion

*Anisakis* infection can cause different types of disease: gastrointestinal anisakiasis, ectopic anisakiasis, gastro-allergic anisakiasis, and specific IgE-positive asymptomatic type [16]. Gastric anisakiasis causes acute abdominal pain a few hours after ingesting infected seafood, and the mechanism of the occurrence of abdominal symptoms remains unclear. Physical irritation from *Anisakis* larval penetration or type I and/or type III allergic reactions were considered one of the causes [17]. In recent years, animal studies using rats have reported that *Anisakis* larvae causes hemorrhages in gastric tissue and mixed inflammatory cell infiltration in neutrophils and macrophages [18, 19]. In addition, proinflammatory cytokines and miRNAs have been investigated [18]; however, their mechanisms remain unknown, which is an issue for the future.

In this study, a relatively large number of asymptomatic cases (22.2%) were found, and there may be more undiagnosed asymptomatic *Anisakis* cases in clinical practice than we think. Moneo et al. indicated that the high number of specific IgE-positive individuals suggests that many asymptomatic patients remain underdiagnosed in endemic countries and that there may actually be more *Anisakis* infections [16]. Although the location of *Anisakis* was not significantly associated with symptoms in previous reports [20, 21], the greater curvature of the upper or middle third stomach was the most common site of penetration, and careful observation of this area is essential for diagnosing gastric anisakiasis.

Univariate analysis revealed significant differences in age, sex, liver cirrhosis, diabetes mellitus, presence or absence of mucosal atrophy, and mucosal atrophy of the penetrating area. Liver cirrhosis showed a subtle result because of the minimal number of patients with positive results. Diabetes mellitus was more common in the asymptomatic group. Elevated pain thresholds have been reported in patients with diabetic neuropathy [22]. Although their severity has not been investigated, patients with diabetes mellitus may be less likely to experience symptoms. Diabetes mellitus was not a significant factor in the multivariate analysis, and this may be associated with the age difference between the two groups. In general, the prevalence of diabetes mellitus increases in older adults [23], and age may have been a confounding factor in our analysis. Multivariate analysis revealed that age, sex, and presence or absence of mucosal atrophy were independent factors for the occurrence of symptoms. The elderly and male patients were significantly more common in the asymptomatic group. This may be because pain thresholds may differ by age and sex. Although there are various opinions about pain thresholds, it has been reported that pain thresholds increase with age, and males may have higher pain thresholds than females depending on the type of pain [24, 25]. Gastric mucosal atrophy was also significantly associated with abdominal symptoms; however, mucosal atrophy of the penetrating area was not an independent risk factor. These results suggest that the presence or absence of gastric mucosal atrophy, rather than mucosal atrophy of the penetrating area, is associated with the occurrence of symptoms. Furthermore, this study found that advanced mucosal atrophy was less likely to cause symptoms. Gastric mucosal atrophy is associated with intragastric pH, and mucosal atrophy results in elevated pH [26]. *Anisakis simplex* is more active at lower pH values and its penetration rate into agar gel increases with decreasing pH [11, 27]. It has been shown that symptoms disappear as *Anisakis* activity decreases [28], suggesting that differences in intragastric pH may have led to differences in *Anisakis* activity and contributed to the differences in symptom occurrence, although the detailed mechanism of abdominal pain remains unclear.

Although not all cases could be examined, there were significant differences in *H. pylori* status, WBC count, and percentage of eosinophils between the two groups. *H. pylori* infection leads to gastric mucosal atrophy. Because gastric mucosal atrophy was significantly associated with symptoms in this study, it seems consistent that the *H. pylori* infection status is indirectly associated with the occurrence of symptoms. In the symptomatic group, the WBC count was above the normal limit in approximately half of the patients. In contrast, all patients in the asymptomatic group had WBC counts within the normal limit. Although leukocytosis was infrequently observed in a previous report [4], our results suggest that the symptoms are accompanied by inflammation. However, in the asymptomatic group, the timing of infection by *Anisakis* was unknown, and the possibility that the WBC count was elevated immediately after infection cannot be excluded. The CRP level may not have elevated because of the short duration from the occurrence of symptoms. Eosinophilia has been reported to be infrequent in gastric anisakiasis cases [4], consistent with the present study, wherein eosinophilia was less frequent in the symptomatic group. Meanwhile, the percentage of eosinophils was frequently elevated in the asymptomatic group; however, the number of patients was too small, making the interpretation of these results difficult.

This study had several limitations. First, this study was a retrospective design, and we could not investigate the *H. pylori* infection status or laboratory data in all cases. In addition, it was impossible to search in detail whether there were any symptoms in asymptomatic cases (for example, patients may have experienced mild symptoms some time ago). In addition, we did not investigate whether abdominal symptoms improved after the removal of *Anisakis* larvae in patients in the symptomatic group. Second, there was selection bias. These results may differ when comparing many cases because there may be more undiagnosed asymptomatic cases. Third, histological examination and molecular analysis of the removed *Anisakis* larvae were not performed. In Japan, *Anisakis simplex* is the major etiological agent of human anisakiasis [29], although different species of *Anisakis* are causative depending on the region. However, a previous study suggested that survival rates in gastric juice or the larval penetrating activity varies among species and that they express genes involved in pathogenicity in a different manner [30–32]. The lack of a species molecular identification is an important limitation, and future analyses with species considerations are needed.

In conclusion, this is the first study to compare the risk factors for acute abdominal symptoms induced by gastric anisakiasis. Age, sex, and presence or absence of mucosal atrophy were the clinical factors associated with the occurrence of symptoms. Older and male patients or those with gastric mucosal atrophy were less likely to show acute symptoms, suggesting that some cases might not have been diagnosed with gastric anisakiasis. However, the mechanisms of symptom occurrence remain unclear and we believe that this study’s results will help clarify this issue.

## Data Availability

The datasets used and/or analyzed during the current study are available from the corresponding author on reasonable request. In the future we may consider asking our patients for their permission to share clinical data for research purposes.

## List of abbreviations

EGD: Esophagogastroduodenoscopy
*H. pylori*: *Helicobacter pylori*
NSAIDs: non-steroidal anti-inflammatory drugs
WBC: white blood cell
CRP: C-reactive protein
RAC: regular arrangement of collecting venules
